# Discovery and characterization of selective lipase-inhibiting polyheterocyclic derivatives: a combined in silico and in vitro study

**DOI:** 10.55730/1300-0152.2748

**Published:** 2025-01-06

**Authors:** Madjda BENGUECHOUA, Mebarka Imene BENGUECHOUA, Khedidja BENAROUS, Houssem BOULEBD, Ibtissem KADI, Arif MERMER, Yakup ŞİRİN, Alaeddine KAOUKA, Mohamed YOUSFI

**Affiliations:** 1 Fundamental Sciences Laboratory, Faculty of Sciences, Amar Telidji University, Laghouat, Algeria; 2 Applied Sciences and Didactics Laboratory, Higher Normal School, Laghouat, Algeria; 3 Department of Chemistry, Faculty of Exact Sciences, University of Constantine 1, Constantine, Algeria; 4 Laboratory of Synthesis of Molecules with Biological Interest, Faculty of Sciences, Mentouri Constantine University, Constantine, Algeria; 5 Department of Biotechnology, Faculty of Health Sciences, University of Health Sciences, İstanbul, Turkiye; 6 Experimental Medicine Application and Research Center, Validebağ Research Park, University of Health Sciences, İstanbul, Turkiye; 7 Department of Pharmacy, Faculty of Health Sciences, University of Health Sciences, İstanbul, Turkiye; 8 Research and Development Center, Semas Food Industry Trade Co. Ltd., Ankara, Turkiye

**Keywords:** Schiff base derivatives, lipase inhibition, in vitro, in silico, ADMET, molecular docking

## Abstract

**Background/aim:**

Obesity has become a global health crisis with an increasing prevalence, necessitating the search for effective therapies. Schiff base derivatives, known for their broad pharmacological activities, have gained attention as potential antiobesity agents. This study aimed to investigate the lipase inhibitory potential of novel Schiff base derivatives and assess their drug-like properties through in vitro assays and in silico methods.

**Materials and methods:**

The lipase inhibitory activity of synthesized Schiff base derivatives was evaluated using in vitro assays, with IC_50_ values determined for each compound. Additionally, in silico ADMET predictions (absorption, distribution, metabolism, excretion, and toxicity), molecular docking studies, and density functional theory (DFT) calculations were conducted to assess the pharmacokinetic properties and binding potential of the compounds to the lipase active site.

**Results:**

The synthesized Schiff base derivatives demonstrated significant lipase inhibitory activity, with IC_50_ values of 995.74 ± 0.010 μM (**6**) and 1985.51 ± 0.041 μM (**2**), comparable to the reference compound quercetin (843.06 ± 0.0007 μM). In silico ADMET analyses revealed that compounds **2** and **6** possess favorable pharmacokinetic properties and exhibit drug-like characteristics. Molecular docking studies showed robust binding interactions between these compounds and the lipase active site, which were further corroborated by DFT calculations that identified reactive regions and stable conformations. Among the compounds, compound **6** exhibited the most effective inhibition and interaction profile, indicating its potential as a lipase inhibitor. These findings underscore the potential of Schiff base derivatives as promising antiobesity agents.

**Conclusion:**

The results of our study highlight the potential of Schiff base derivatives as promising candidates for antiobesity therapy, given their significant lipase inhibitory activity and favorable in silico predictions. Further research is needed to elucidate the precise mechanisms of action and assess the efficacy of these compounds in vivo.

## 1. Introduction

The prevalence of overweight and obesity has risen at an alarming rate in recent decades, particularly among children and adolescents, and is one of the greatest public health challenges of the twenty-first century (Thibault and Rolland-Cachera, 2003). Intergenerational causes interacting with lifestyle factors such as dietary changes or lower levels of physical activity are well documented risk factors for obesity (Popkin et al., 2012). Over 390 million children and adolescents aged 5–19 years were overweight in 2022. The prevalence of overweight (including obesity) among children and adolescents aged 5–19 rose dramatically from just 8% in 1990 to 20% in 2022. The rise has occurred similarly among both boys and girls; in 2022, 19% of girls and 21% of boys were overweight (Aissaoui et al., 2023). Obesity is an important global health problem that affects various countries, particularly Algeria, where overweight and obesity are becoming a serious public health problem. One in two Algerians and one in three Algerian women are overweight (Kumar and Kelly, 2017).

Drug discovery begins when there are no appropriate drugs for a specific disease or clinical condition. In this scenario, the pharmaceutical industry and academic research groups undertake separate processes to identify new molecules with drug-like characteristics that interact effectively with the intended biological target. These new chemical entities may come from natural sources or be synthesized through chemical techniques (Atanasov et al., 2021).

Synthetic molecules are produced by total synthesis, offering the possibility of designing compounds with specific properties for various applications. These molecules play a central role in improving human health, diagnostics, research, and our understanding of living organisms. The biological activity of a synthetic compound can vary greatly depending on its specific structure and properties (Khan et al., 2023; Ayaz et al., 2024).

Schiff bases, also known as imines or azomethines, have gained considerable interest due to their wide range of applications (Okey et al., 2020). Schiff bases and their derivatives are further recognized for their diverse pharmacological properties, encompassing antibacterial, antifungal, antioxidant, antiinflammatory, antitumor, anticancer, and antimicrobial activities (Pontiki et al., 2008; Kumar et al., 2010; Da Silva et al., 2011; Amer et al., 2013; Bensaber et al., 2014; Güngör and Gürkan, 2014; El-Wakiel et al., 2015; Shanty et al., 2017; Hussain et al., 2023; Khan et al., 2025). Additionally, Schiff bases play roles in biological systems and as intermediates in enzymatic reactions (Yeap et al., 2003).

The aim of our study was to investigate the lipase inhibitory potential of synthesized Schiff base derivatives and evaluate their drug-like properties. In vitro enzyme assays and in silico ADMET predictions were utilized to assess their efficacy and potential for drug development. Molecular docking studies were conducted to elucidate the binding interactions between these compounds and the lipase active site. These in silico approaches have significantly accelerated drug discovery by identifying compounds with high binding affinities and desirable drug-like properties (Alam et al., 2022; Khan et al., 2022).

## 2. Materials and methods

### 2.1. Chemicals and reagent

*Candida rugosa* lipase, p-nitrophenyl laurate, and all other reagents used in the synthesis, purification, and biological activity were purchased from Sigma Aldrich. All other chemicals and solvents used were of analytical grade with a high level of purity suitable for laboratory and analytical applications (purity of at least 95%).

### 2.2. Synthesis

Compounds 1–7 were prepared as described in our previous studies (Kadi et al., 2023, 2024a, 2024b).

### 2.3. In vitro lipase assay

The method used to evaluate the effectiveness of these synthetic compounds against *C. rugosa* lipase (CRL) in vitro followed the same procedures outlined in our previous studies (Benarous et al., 2013; Benguechoua et al., 2014; Nia et al., 2014; Benarous et al., 2015, 2017; Serseg and Benarous, 2018; Nebeg et al., 2019; Serseg et al., 2020, 2024) with quercetin as a reference compound. Briefly, p-nitrophenyl-laurate (p-NPL) was used as a substrate, while p-nitrophenol (p-NP) was utilized as the standard for creating a calibration curve. The activity of one unit was determined as the enzyme quantity that liberated 1 μmol of p-NP per minute following the specified assay conditions. The IC_50_ values, representing the concentrations at which 50% inhibition of lipase activity occurs, were determined through regression analysis of the inhibition versus synthetic compound concentration curves. All experiments were done at least in triplicate.

### 2.4. In silico approach

#### 2.4.1. Computational chemistry

##### 2.4.1.1. Density functional theory (DFT) calculations

The molecular geometry, chemical reactivity, and intramolecular interactions of compounds 2 and 6, which showed the most promising biological activity, were determined by quantum chemical calculations. The DFT method at the theoretical level B3LYP/6-31+G(d,p) was selected for all calculations. This theoretical level offers a good balance between accuracy and resource consumption, making it a frequent choice in several previous studies (Tirado-Rives and Jorgensen, 2008; Niknam et al., 2021; Abad et al., 2023; Waheed et al., 2023). The fundamental states were validated by verifying the absence of imaginary frequencies. The isosurfaces of Fukui functions (*f^+^* and *f* ^−^) and dual descriptors (Δ*f*), as well as the noncovalent interactions based on the reduced density gradient (NCI-RDG) analysis were performed using the software Multiwfn and visualized with VMD (Humphrey et al., 1996; Lu and Chen, 2012). All calculations were performed using the software Gaussian 09 (Frisch et al., 2009).

#### 2.4.2. Prediction of biological activities

The online tool PASS was used to assess the potential biological activities of the most effective inhibitors identified in vitro, specifically two synthetic compounds of Schiff base derivatives. Both compounds were generated using the software package ChemOffice version 2016. Subsequently, the canonical SMILES were copied and pasted into the PASS online server. This predictive method relies solely on a structural activity relationship (SAR) analysis of a training set comprising more than 205,000 chemicals with diverse biological activities, encompassing over 3750 variations (Khurana et al., 2011; Garg et al., 2021; Linani et al., 2022; Bellahcene et al., 2024).

#### 2.4.3. Assessment of drug-likeness (ADMET)

ADMET, an acronym standing for absorption, distribution, metabolism, excretion, and toxicology, serves as a cornerstone in drug development. It focuses on understanding how a drug travels through the body, interacts with organs, and is ultimately eliminated. By examining these factors, scientists can predict a drug’s effectiveness and potential for side effects (Cheng et al., 2012).

Understanding a drug’s ADMET properties is crucial in medicine, as they directly impact how well a medication works and how safe it is for patients. Accurately predicting these properties early in development is essential for streamlining the drug development process and ensuring the delivery of effective and safe medications (Guan et al., 2019). In the present study, we tested the ADMET properties of the two synthetic compounds of Schiff base derivatives to examine their potency in being a good drug or not. We evaluated the ADMET properties of the synthetic compounds 2 and 6, through various online platforms such as pre-ADMET v2.0 (Lee et al., 2002), a server that has been recommended and cited in the literature (Szymański et al., 2011). AdmetSAR 2.0^1^ (Yang et al., 2019) and the SwissADME server^2^ (Daina et al., 2017) were used.

#### 2.4.4. Molecular docking

Docking simulations were conducted using the same methodology as described in our previous studies (Benarous et al., 2018, 2021; Serseg et al., 2021, 2022). Ligands were obtained from the PubChem database, and a lipase structure (1LPA) was retrieved from the Protein Data Bank. Protein preparation involved removing water, heteroatoms, and ligands, followed by the addition of polar hydrogens and Gasteiger charges. Docking simulations were performed using AutoDock Vina (Liu et al., 2006), with binding box dimensions defined using ADT. The default AutoDock Vina parameters were used, except for the number of output conformations, which was set to 1. Docking results were analyzed in Discovery Studio Visualizer to determine the preferred conformation and type of inhibition. The active site of 1LPA was identified as previously reported (Batubara et al., 2009).

## 3. Results and discussion

### 3.1. Synthesis

### 3.2. In vitro lipase assay

Schiff base derivatives are attracting considerable attention in drug development and other biological fields due to their potential applications (Kajal et al., 2013). In the present study, the potential inhibitory activity of lipase in vitro and in silico of Schiff base derivatives was examined.

**Figure 1:**
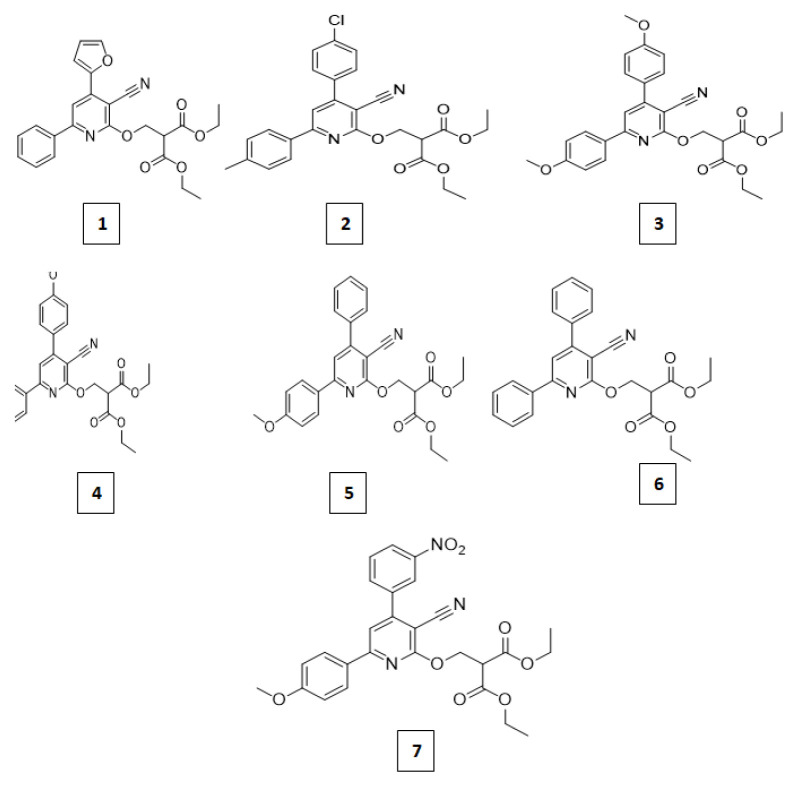
The structure of the synthesized Schiff base derivatives **1**–**7**.

To produce biologically active compounds, a range of acylating agents were carefully chosen; the present paper outlines the synthesis of seven Schiff base derivatives with various substituents (Figure 1).

**Figure 2:**
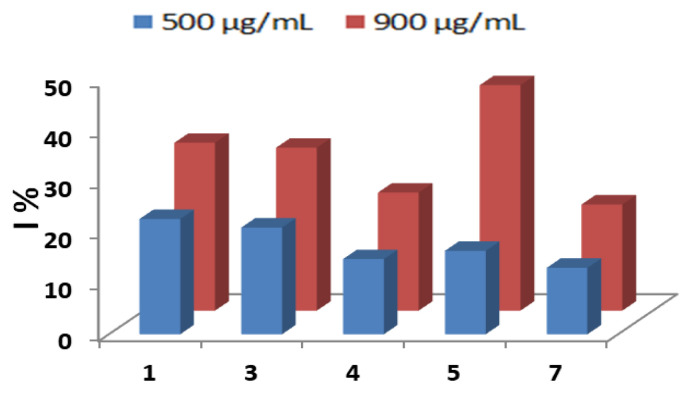
Histogram of lipase inhibition percentages by Schiff base derivatives.

**Figure 3:**
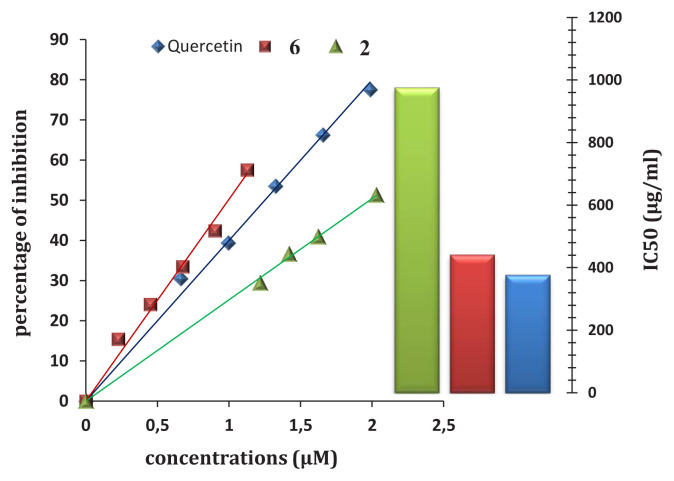
Inhibition curves and values of lipase by Schiff bases and quercetin.

The IC_50_ values were calculated from the plot of the enzyme activity as a function of synthetic compound concentration. The synthetic compounds 2 and 6 of Schiff base derivatives presented the highest inhibitory activity against lipase with IC_50_ = 1985.51 and 995.74 μM respectively, while the other synthetic compound showed weak activities (Figure 2); the synthetic compound **6** showed the best inhibition with IC_50_ = 995.74 μM. Thus, compound **7** showed the lowest inhibition (PI ≤ 30); comparing these results to quercetin, both synthetic compounds are less potent than quercetin with IC_50_ of 843.06 μM (Figure 3). It is likely that the varying inhibitory effects of these synthetic compounds are due to their distinct chemical structures and physicochemical properties. Significant effort has been dedicated to discovering novel and potent lipase inhibitors derived from natural sources that have fewer side effects and can be used to treat various conditions, such as acne, candidiasis, and obesity (Benarous et al., 2018).

To assess the effectiveness of our synthesized compounds, we compared their lipase inhibitory activity against that of previously reported inhibitors. Chloramphenicol, with an IC_50_ value of 220 ± 0.003 μg/mL (Liu et al., 2006), exhibits greater potency as an antiacne medication compared to the synthetic compounds we investigated. However, tetracycline (IC_50_ = 470 ± 0.005 μg/mL) (Liu et al., 2006) and loratadine (IC_50_ = 440 ± 0.02 μg/mL) (Benarous et al., 2018) demonstrate similar efficacy to the synthetic compound **6**. Considering this, a combination therapy involving **6** along with other medications could be considered as a potential treatment option for managing acne effectively. Molecules isolated from natural extracts have demonstrated inhibitory activity against lipase, for instance, harmaline, hispidin, and catechin, with IC_50_ = 830, 179, and 183 μg/mL, respectively (Ruiz et al., 2006; Benarous et al., 2015). In order to compare our studied synthetic compound with other lipase inhibitors, we found that our compound **6** is more potent than folic acid and febuxostat with IC_50_ values of 0.64 and 0.66 mg/mL, respectively (Serseg and Benarous, 2018).

### 3.3. In silico approach

#### 3.3.1. Computational chemistry

##### 3.3.1.1. DFT calculations

**Figure 4:**
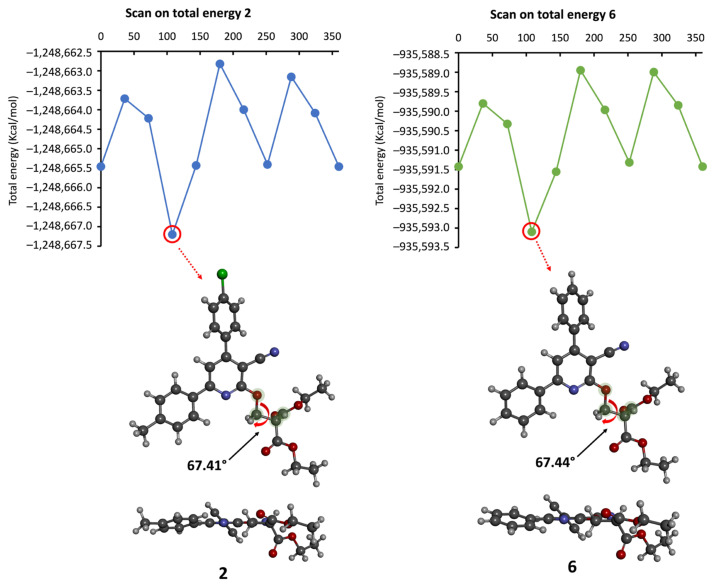
Scan of the total energy of compounds **2** and **6** computed at B3LYP/6-31+G(d,p).

The molecular geometry of the compounds that showed the most promising results (2 and 6) was analyzed using DFT calculations at the theoretical level B3LYP/6-31+G(d,p). Since the two aromatic groups linked to the pyridine ring are involved in π-electron delocalization, they cannot perform rotations. In contrast, the malonate group is flexible and able to rotate. Thus, a rotational scan study was first carried out for both compounds 2 and 6, focusing on the potential rotation of the malonate group. Nine steps, with an interval of 36°, were examined as illustrated in Figure 4. It emerged from this analysis that the most stable rotamer for compounds 2 and 6 corresponds to a dihedral angle (O–CH_2_–CH–C=O) of 67.41° and 67.44°, respectively. This rotamer is around 2 to 4 kcal/mol more stable than the others, suggesting that it dominates for both derivatives 2 and 6.

**Figure 5:**
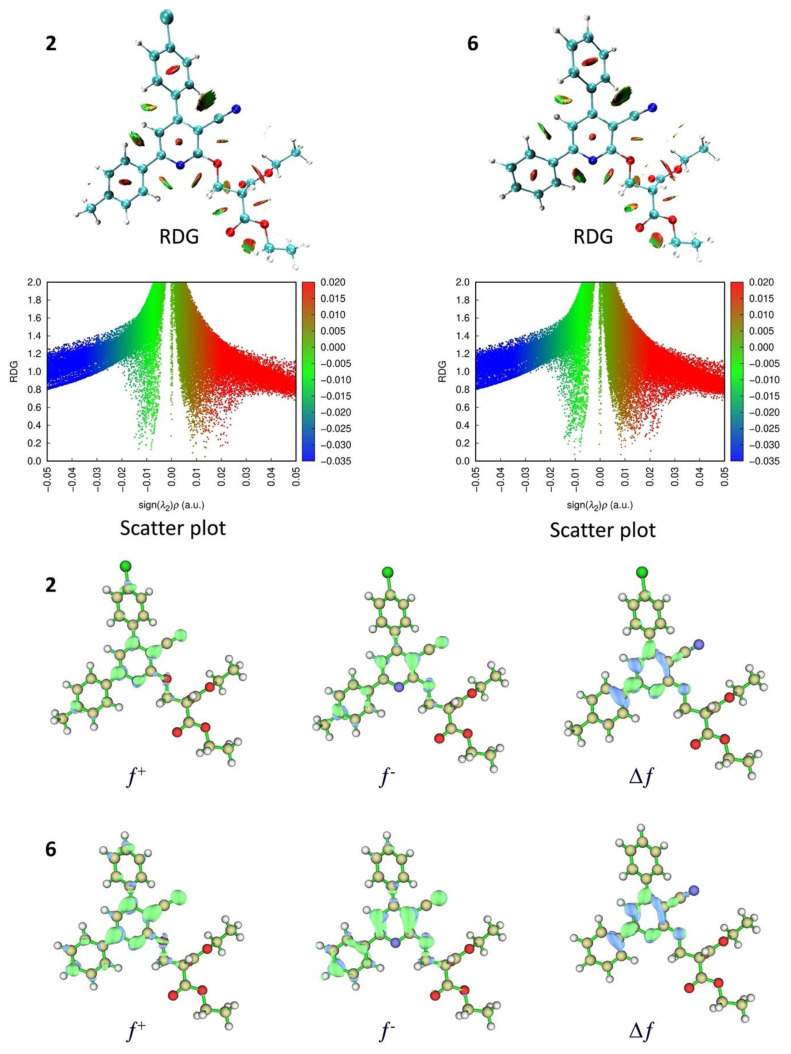
Computed RDG isosurface maps, scatter plots, Fukui functions, and dual descriptors of compounds **2** and **6** computed at B3LYP/6-31+G(d,p).

The Fukui functions *f ^+^* and *f* ^−^, as well as the dual descriptors Δ *f*, were calculated for both molecules 2 and 6 and are visualized as isosurface maps in Figure 5. The Fukui functions *f* ^+^ and *f* ^−^ denote the sites most likely to react with nucleophiles and electrophiles, respectively, while the dual descriptors highlight the most reactive regions of a molecule (Singh et al., 2023). As illustrated in Figure 5, *f ^+^* and *f* ^−^ show that the pyridine ring, the cyano group, and the phenyl ring at position 6 are both the main nucleophilic and electrophilic sites, the difference residing solely in the constituent atoms of these groups. Of these regions, the pyridine ring and cyano group are the most reactive sites, defining the overall reactivity of the molecules, as indicated by the Δ*f*.

The intramolecular interactions of compounds 2 and 6 were studied using the noncovalent interaction (NCI) method, based on the reduced density gradient (RDG) (Johnson et al., 2010; Jan et al., 2024). The RDG isosurface map and corresponding scatter plot are shown in Figure 5. The two molecules reveal several intramolecular contacts, both repulsive and attractive. Among these interactions, four stand out for their importance, represented as green areas on the RDG isosurface map and by a peak near the zero point in the scatter plot. These contacts are all of the van der Waals type, with the most significant interaction observed between the phenyl ring and the CN group.

#### 3.3.2. Prediction of biological activities

We utilized the PASS web server^3^ to forecast the biological activities (BA) of the most promising compounds, specifically compounds 2 and 6. The PASS results, as best Pa and Pi, are summarized in Table 1. We observed that compound 2 exhibited 197 BA within a Pa range of 0.636 to 0.042. This synthesized compound demonstrated varied potential activities such as lipoprotein lipase stimulant (Pa = 0.241) and glycosylphosphatidylinositol phospholipase D (Pa = 0.319).

For compound 6, we saved 372 BA with Pa values ranging from 0.592 to 0.02. We discovered diverse effects of compound 6, such as glycosylphosphatidylinositol phospholipase D inhibitor (Pa = 0.380) and lipoprotein lipase and stimulant with Pa = 0.250 and 0.197, respectively, and as phosphoinositide phospholipase C inhibitor (Pa = 0.124).

Based on these results, both compounds could be tested in vitro for enzymatic inhibition of lipase to increase their chances of being used as drug candidates for the treatment of diseases.

#### 3.3.3. Assessment of drug-likeness (ADMET)

The results of the ADMET drug likeness evaluation are summarized in Table 2. This procedure anticipates the characteristics of a medication by examining its chemical composition synthetic compounds with specific properties that play a crucial role in the development of new drugs. The key properties of a synthetic compound that are essential for the development of safe and effective drugs, such as toxicological potential, drug–drug interaction potential, metabolic stability, and intestinal permeability, could be selected to use to develop new safe and effective drugs (Li, 2001). According to the results given in Table 1, the 2 and 6 molecules conformed to Lipinski’s rule, meaning that both molecules were predicted to be orally bioavailable, that they have positive human intestinal absorption; water solubility depends on the nature of the structure, temperature, and concentration. BBB < 1 indicates that the compounds easily cross the blood–brain barrier. Cytochrome P450 values show the probability of being inhibitors and substrates. The different routes of excretion of the two compounds indicate high elimination from the body. Both compounds were expected to be toxic and interacted with other targets.

#### 3.3.4. Molecular Docking

**Figure 6:**
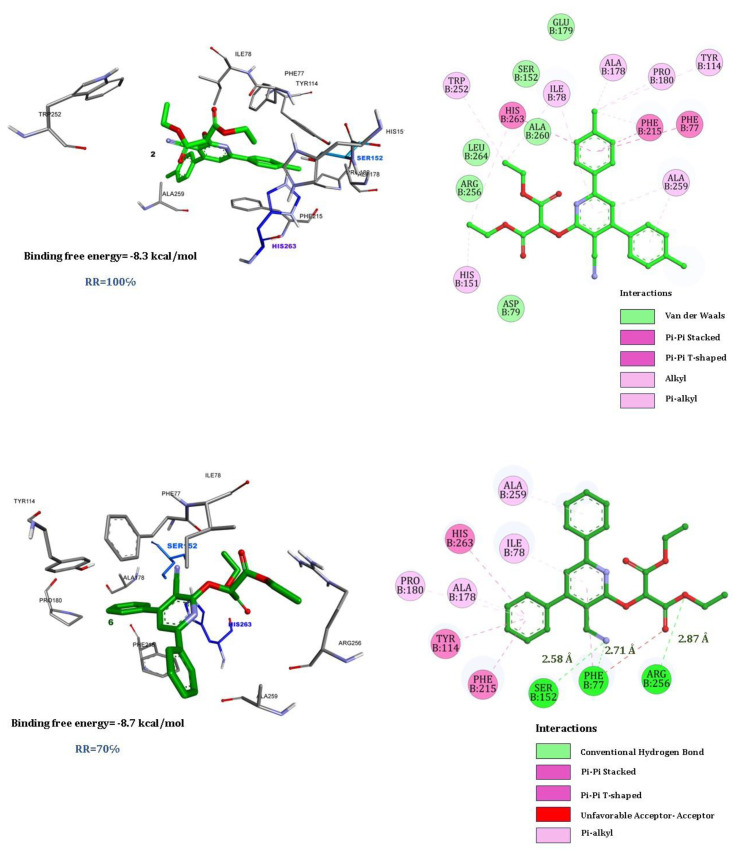
Best docking poses for the three compounds, **2**, **6**, and quercetin.

Figure 6 shows the results of a molecular docking study involving two synthesized compounds, quercetin with human pancreatic lipase. The results are visually represented in two main parts: a 3D model of the binding interaction and a 2D interaction diagram.

The docking simulation predicts a binding free energy of −8.3 kcal/mol for compound 2 (Figure 6a), indicating a favorable interaction between the compound and the enzyme. This compound is shown interacting with several amino acids within the binding pocket of human pancreatic lipase. Notable residues involved include two AA from the catalytic triad SER152 and HIS263, and other as HIS15, PHE215, and ALA259. Van der Waals forces are predominant, involving multiple contacts throughout the binding site, which help in positioning the compound stably within the lipase. Pi–pi interactions (both stacked and T-shaped) involve aromatic amino acids, which are crucial for the binding specificity and stability. Alkyl and pi–alkyl interactions also contribute to the hydrophobic bonding within the pocket.

Compound 6 (Figure 6b) provided a slightly more favorable binding free energy of −8.7 kcal/mol compared to the previous compound 2. We noted conventional hydrogen bonds between the compound and amino acids like SER52 (2.52 Å), ARG256 (2.87 Å), and PHE77 (2.77 Å), critical for stable molecular interactions. The pi–pi stacked interactions likely occur with aromatic residues such as PHE215 and TYR114, which increase the stability of this complex. For pi–pi T-shaped interactions, they typically involved residues like His263 and Phe77. Pi–alkyl suggested interactions between the aromatic parts of the ligand and the alkyl side chains of amino acids as ILE78, ALA178, and ALA259, which provide additional stability.

The binding free energy of quercetin (Figure 6c) as an inhibitor of human pancreatic lipase is −9.8 kcal/mol. This value suggests a strong binding affinity between quercetin and the enzyme, indicating its potential effectiveness as an inhibitor. The residues ARG256, PHE215, and SER152 formed hydrogen bonds with quercetin with these values respectively (2.42, 2.25, 2.35), strengthening the binding through polar interactions.

The mix of hydrogen bonding, pi–pi interactions (both stacked and T-shaped), and hydrophobic interactions (pi–alkyl) contribute significantly to the stability and specificity of the compound’s binding to human pancreatic lipase.

Overall, the docking results suggest a strong and specific binding of the synthesized compounds 2 and 6 to human pancreatic lipase, potentially influencing the enzyme’s activity. Such interactions are critical for the design of inhibitors that can modulate the function of pancreatic lipase, possibly for therapeutic applications such as the management of hyperlipidemia.

## 4. Conclusion

The present study introduces Schiff base derivatives as promising lipase inhibitors with potential applications in antiobesity therapy. By combining in vitro enzymatic assays with in silico ADMET and docking studies, it demonstrates significant inhibitory activity and favorable pharmacokinetic profiles. The novelty lies in the synthesis of polyheterocyclic compounds with dual functional properties, offering a strong foundation for designing targeted antiobesity agents. Unlike existing inhibitors, these compounds exhibit unique binding interactions and structural adaptability for optimization. Future in vivo studies are essential to translate these findings into therapeutic applications

## Figures and Tables

**Table 1:** The predicted biological activities for the two best compounds, **2** and **6**.

Compounds	Pa	Pi	PBA
**2**	0.241	0.029	Lipoprotein lipase stimulant
	0.319	0.2	Glycosylphosphatidylinositol phospholipase D inhibitor
**6**	0.380	0.157	Glycosylphosphatidylinositol phospholipase D inhibitor
	0.250	0.150	Lipoprotein lipase inhibitor
	0.197	0.097	Lipoprotein lipase stimulant
	0.124	0.095	Phosphoinositide phospholipase C inhibitor

**Table 2:** ADMET drug likeness evaluation of synthesized compounds **2** and **6**.

	Molecule	2	6

Medicinal Chemistry	Lipinski	+	+

	Pfizer Rule	+	+

ABSORPTION	Human Intestinal Absorption	+(0.005)	+(0.004)

	Caco-2	+(−4.663)	+(−4.618)

Distribution	Water solubility (mg/ml)	2.9.10^−4^	2.11.10^−3^
	BBB Penetration	+(0.066)	+(0.3)
	Volume Distribution (l/kg)	0.472	0.604

METABOLISM	CYP450 1A2 Inhibitor	+	+

	CYP450 2C9 Inhibitor	+	+

	CYP450 2D6 Inhibitor	−	−

	CYP450 2C19 Inhibitor	+	+

	CYP450 3A4 Inhibitor	+	+

TOXICITY	AMES Toxicity (probability)	+(0.148)	+(0.103)

	Carcinogens (probability)	+(0.163)	+(0.211)

	Hepatotoxicity (probability)	−(0.882)	− (0.841)
	Drug induced liver injury	− (0.967)	− (0.975)
	Skin Sensitization	+(0.071)	+(0.115)
	Eye Irritation	+(0.246)	±(0.514)
	Maximum recommended Daily Dose	+(0.104)	+(0.062)
	Respiratory Toxicity	+(0.045)	+(0.049)

(+): safe; (−): dangerous; **(±)**: medium risk

## Footnotes

1 ADMETSAR (2023). ADMET Structure-Activity Relationship Database [online]. Website http://lmmd.ecust.edu.cn/admetsar2/ [accessed 11 September 2024]

2 SwissADME (2023). SwissADME: A Free Web Tool to Evaluate Pharmacokinetics, Drug-Likeness, and Medicinal Chemistry Friendliness [online]. Website http://www.swissadme.ch/index.php [accessed 11 September 2024]

3 PASS Online (2014). Prediction of the biological activity spectra of organic compounds using the PASS online web resource [online]. Website http://www.pharmaexpert.ru/passonline/ [accessed 11 September 2024]
